# Subarachnoid haemorrhage secondary to traumatic intracranial aneurysm of the posterior cerebral circulation: case series and literature review

**DOI:** 10.1007/s00701-016-2865-6

**Published:** 2016-06-30

**Authors:** Ruth-Mary deSouza, Munirih Shah, Panayiotis Koumellis, Mansoor Foroughi

**Affiliations:** 1 Department of Neurosurgery, Brighton and Sussex University Hospitals NHS Trust, Eastern Rd, Brighton, East Sussex BN2 5BE UK; 2 Department of Neurosurgery, King’s College Hopsital, Denmark Hill, London, SE5 9RS UK

**Keywords:** Subarachnoid, Haemorrhage, TICA, Posterior circulation, Trauma, Pseudoaneurysm, Delayed

## Abstract

**Background:**

To identify the clinical features, rebleed risk, timing and method of diagnosis, complications and outcome for subarachnoid haemorrhage (SAH) from traumatic intracranial aneurysm (TICA) of the posterior circulation. Subjects included 26 patients aged 3–54 (mean 24.8).

**Methods:**

Case series and literature search to identify all reported cases.

**Results:**

In our series, two of three cases were fatal as a result of rebleed, and one case had a good outcome with no deficit, following prompt diagnosis and embolisation. Our key findings from the literature review were: 30.7 % of patients were age 16 and under; 88 % had an acute drop in consciousness, 46 % in a delayed manner; the mean time to diagnosis was 7.5 days; initial cerebrovascular imaging was normal in 23 %; the rebleed rate was 23 %; 61 % required emergency diversion of cerebrospinal fluid; 11 % developed vasospasm requiring treatment; 19.2 % had deficits that rendered them unable to live independently. The mortality rate was 27 %.

**Conclusions:**

SAH from ruptured posterior circulation TICA is associated with significant morbidity and mortality. A high index of suspicion as well as prompt diagnosis, repeat imaging in selected cases, and treatment of any associated TICA can be crucial to a favourable outcome.

## Introduction

Subarachnoid haemorrhage (SAH) secondary to traumatic intracranial aneurysm (TICA) of the posterior cerebral circulation is a rare but treatable cause of neurological morbidity and mortality in young patients [1]. The significant mortality associated with this condition is likely due to the high risk of rupture and rebleed when compared to berry aneurysms [2]. Given the limited evidence regarding the natural history and complications associated with this diagnosis, and the technical challenges of treating a TICA, currently the management of this condition is on a case-by-case basis. We present a case series and a review of the literature for all reported cases of SAH from posterior circulation TICA. We seek to clarify the clinical features, rebleed risk, timing and method of diagnosis, complications and outcome for this increasingly recognised condition.

## Case 1

**Fig. 1:**
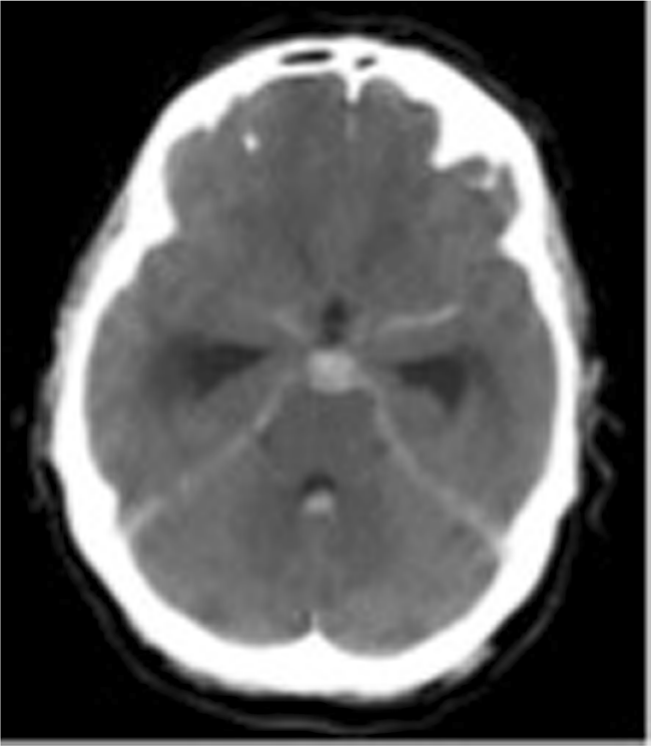
CT brain scan demonstrating subarachnoid blood concentrated in the basal cisterns and fourth ventricle and ventriculomegaly

A 20-year-old male was assaulted via a punch to the face. He fell, hitting his occiput on the ground and losing consciousness. On arrival in the emergency department, he was found to be alcohol intoxicated, and a computed tomophraphy (CT) scan of the brain was performed 6 h post injury. It demonstrated SAH in the basal cisterns, third and fourth ventricles and acute hydrocephalus (Fig. 1). An intracranial computerised tomographic angiography (CTA) did not demonstrate any vascular abnormality. His Glasgow Coma Scale (GCS) score gradually recovered to 15, he had no focal neurological deficit, and he was therefore managed conservatively. Digital subtraction angiography (DSA) at day 3 post injury did not demonstrate any vascular abnormality. At 11 days post presentation, whilst still an inpatient being monitored and considered for delayed imaging, the patient was found collapsed and in cardiac arrest. Resuscitation was unsuccessful. A post mortem demonstrated massive SAH from a vertebral artery TICA following a suspected traumatic dissection.

## Case 2

A 21-year-old male presented with headache and drowsiness 24 h after a head injury due to an assault. On presentation, his GCS was 14 (E4V4M6) and an emergency CT brain scan demonstrated SAH, which was predominantly around the fourth ventricle and pre-pontine cistern with acute hydrocephalus (Fig. panel 2). Over the next 2 h the patient’s GCS dropped to 10 (E3V2M5) and an external ventricular drain (EVD) was placed. Following insertion of the EVD a CTA of the circle of Willis was performed.

**Fig. 2:**
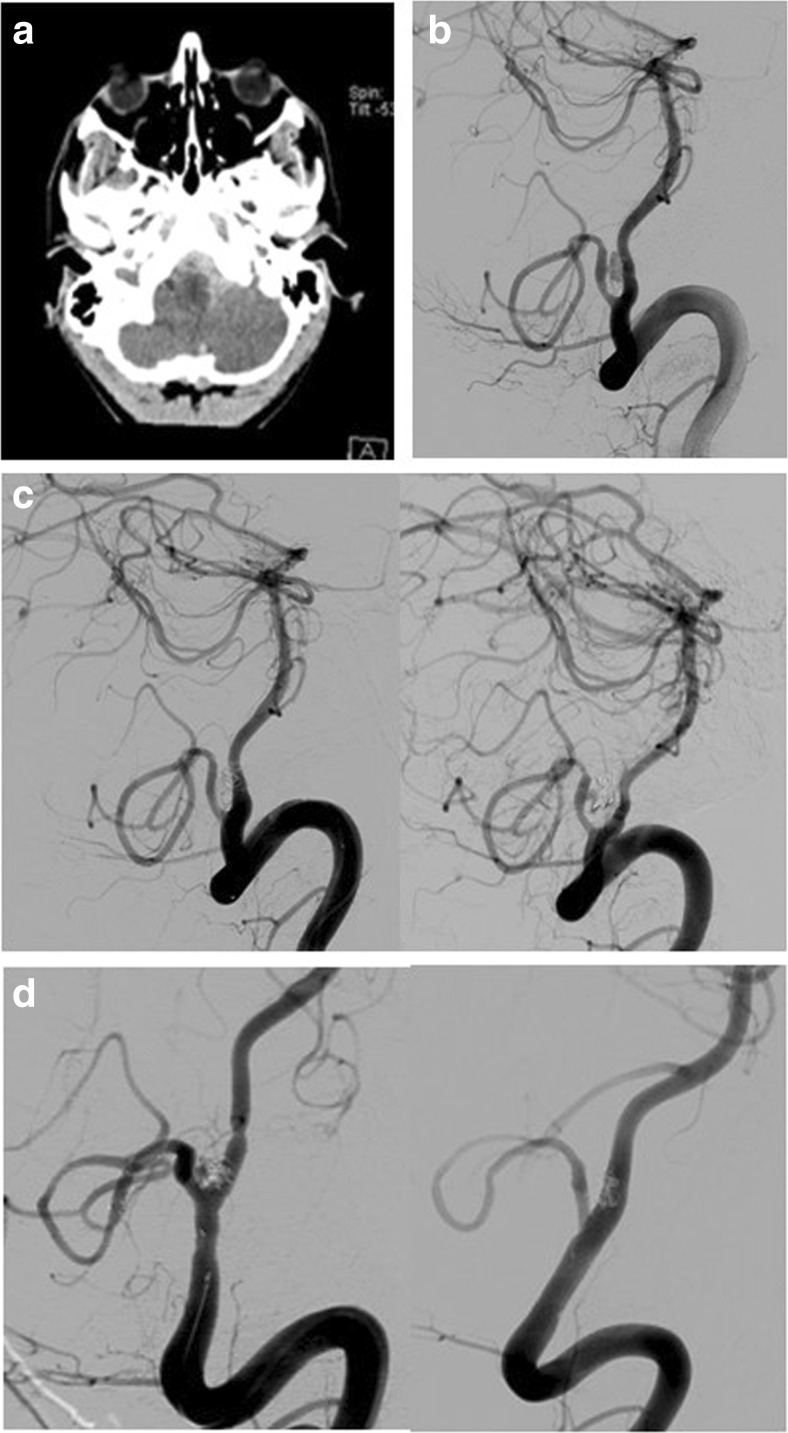
**a** CT head showing SAH in the posterior fossa cisterns, most prominent around the medulla and hydrocephalus. **b** DSA demonstrating traumatic PICA dissection and TICA. **c** A loose coiling of the pseudosac was performed in the first instance (*left*). A DSA was performed 3 days later (*right*) showed an enlarging pseudosac. **d** Two stents were inserted in the left PICA (*left*). At the end of the procedure there was still some filling of the pseudosac. A follow-up DSA was performed 2 months later (*right*) that demonstrated obliteration of the aneurysm and patency of the PICA

The CTA demonstrated abnormal appearances of the left posterior inferior cerebellar artery (PICA), suspicious for a dissection and TICA. The patient underwent a DSA, which confirmed the findings (Figs. panel 2 and 3). Following multidisciplinary discussion the patient underwent endovascular treatment of the TICA, the same day. The pseudosac was loosely coiled with the plan to follow-up closely. The patient woke up with a GCS of 14 (E4V4M6) with no focal deficit.

**Fig. 3:**
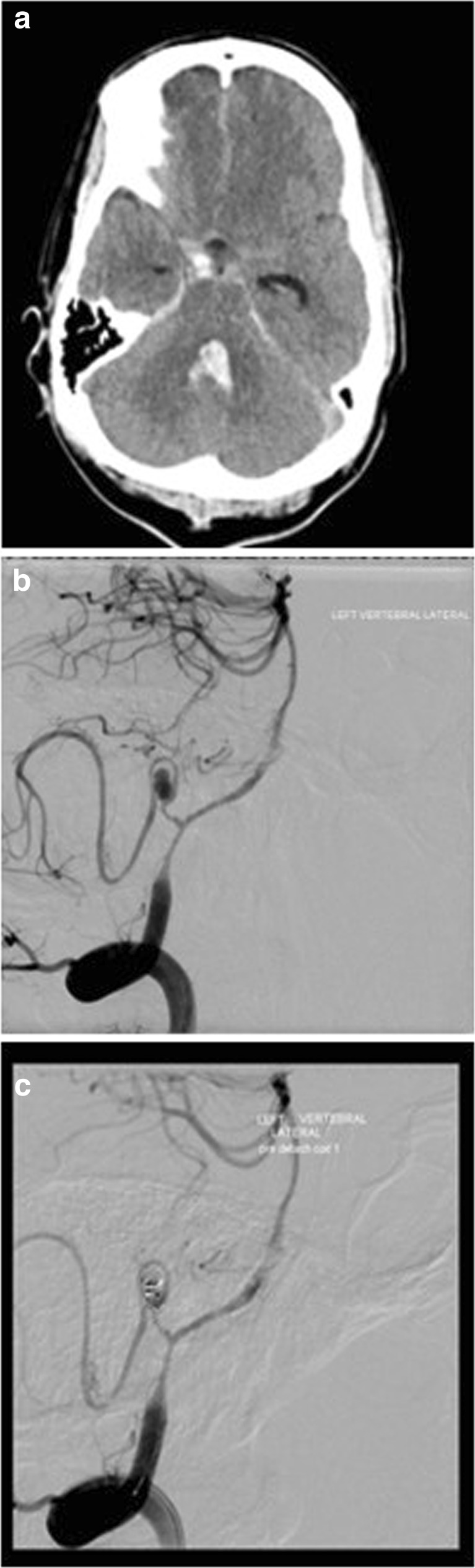
**a** CT demonstrating diffuse subarachnoid blood and intraventricular haemorrhage with hydrocephalus. **b** DSA demonstrating left PICA. **c** Post coiling DSA demonstrating coil occlusion of the left PICA TICA

A follow-up DSA was performed 3 days later, which showed an enlarging pseudosac, and the decision was made to proceed to stenting of the dissected PICA vessel. Two Solitaire stents (Covidien, Irvine, CA) 3 × 20 were placed in the PICA, one inside the other. It was not deemed safe to position more coils in the pseudosac that was arising at a sharp angle from the PICA. The patient woke up with no new neurological deficit and remained stable for the next 24 h.

A DSA at 3 months showed obliteration of the pseudoaneurysm and a patent PICA. A magnetic resonance angiogram (MRA) was performed at 1 year and showed patency of the PICA and no infarct in the posterior circulation. Clinically, the patient was well except for a mild and resolving hemiparesis related to a haematoma associated with EVD removal. The patient did not attend a second follow-up DSA.

## Case 3

A 35-year-old male was assaulted via a punch to the head and hit his head on a pavement, leading to loss of consciousness. He was intubated and ventilated prior to arrival at the neurosurgical department. A CT and CTA brain on admission to the emergency department demonstrated extensive subarachnoid blood throughout the basal cisterns and the hemispheric fissures. Most of the subarachnoid blood was layered around the cervicomedullary junction and the cerebellar tonsils. Blood was noted in all of the ventricles, and acute hydrocephalus was present. An EVD was placed to treat the hydrocephalus. The CTA did not demonstrate any vascular abnormality. The patient was gradually weaned off sedation and recovered to GCS 13/15 (E3V4M6). At 7 days post admission the patient had a second bleed with neurological deterioration and fresh haemorrhage seen in the cerebrospinal fluid (CSF) draining from the EVD. A DSA was carried out and a left PICA dissecting TICA was identified and treated with endovascular coiling.

He subsequently required ventriculo-peritoneal shunt insertion to treat his hydrocephalus and developed complications associated with shunt blockage and CSF infection. He died 3 months later from sepsis and multi-organ failure.

## Results

The literature search, conducted via MEDLINE, PubMed and EmBase, identified 26 cases of SAH secondary to a ruptured posterior circulation TICA. These are summarised in Table 1. The mean patient age was 24.8 (range 3–54 years); 8/26 patients (30.7 %) were age 16 and under. Falls accounted for the trauma mechanism in eight cases (30.7 %), assault to the head in seven (27 %) cases and a contact sport in four (15 %) cases. The majority were blunt trauma, with only three cases being penetrating. In terms of clinical presentation, 23/26 (88 %) had an acute drop in consciousness, either at initial presentation and/or in a delayed manner (12/26, 46 %) with 50 % of these being due to a rebleed. Each patient had a solitary TICA and the vessel involved most commonly was the PICA (14 patients, 54 %), with the left PICA in eight cases (30.7 %) and the right PICA in six (23 %). Other TICAs were of the vertebrals (30.7 %): left vertebral in four cases, right in four cases. The left superior cerebellar artery was involved in two cases (7.7 %) and basilar in two (7.7 %) cases. Time to definitive diagnosis of a TICA was variable, from 1 day to 21 months. Excluding the one case where it was detected at 21 months, the mean time to diagnosis was 7.5 days; 24/26 (92 %) cases had subarachnoid blood on their presenting CT brain scan. One patient had no SAH on the first scan and was neurologically intact, subsequently deteriorating at day 8, when the second CT brain demonstrated haemorrhage [12]. One case was diagnosed by lumbar puncture [6]. In six cases (23 %), there was initially normal cerebrovascular imaging. Repeat imaging was prompted by clinical deterioration or suspicion of an unusual distribution of subarachnoid blood. Excluding the case diagnosed at 21 months [5], the mean time to diagnosis in this group with initially negative vascular imaging was 6.4 days. The diagnosis of TICA was made by CTA in seven cases (27 %), but DSA was required for further characterisation and treatment planning in the majority of cases. In terms of management, 11/26 (42.3 %) were managed surgically, with clipping, bypass and vessel occlusion. Two surgical cases were after a previous endovascular procedure [8, 12]; 10/26 cases (38.5 %) were treated endovascularly (coiling, vessel occlusion, stenting); 5/26 (19.2 %) were unsuitable for treatment because of their severe clinical condition; 16/26 (61.5 %) patients required emergency CSF diversion to treat acute hydrocephalus or for management of intracranial pressure. Fourteen patients had an EVD placed and two required a ventriculoperitoneal shunt. In terms of clinical outcome, seven patients died (mortality 27 %), and five (19.2 %) had deficits that affected their ability to live independently. Ten of 26 (38.5 %) were reported to have made a sufficient recovery to live independently or almost independently. Evaluating complications, rebleed occurred in 6 patients (23 %), hydrocephalus requiring permanent CSF diversion in 14 (53.8 %), vasospasm requiring angioplasty in 3 (11.5 %) and stroke in 2 (7.7 %) patients.

**Table 1:** Summary of the literature review of patients presenting with subarachnoid haemorrhage secondary to traumatic posterior cerebral circulation TICA formation

Author and year	Age	Trauma mechanism	Neurological symptoms	Vessel involved	Timing of TICA diagnosis (days)	SAH on initial scan	Was any negative vascular imaging performed?	Investigations that diagnosed TICA	Management	EVD initially for hydrocephalus or ICP management	Outcome	Complications
Hossain et al., 2002 [3]	12	Fall	Delayed acute drop in conscious level at day 19 due to hydrocephalus and day 21 due to rebleed	Right PICA	21	Yes	No (first CTA was at day 21 after rebleed)	CTA and then DSA at day 21	Coiling	VP shunt as initially no IVH	Right arm weakness that improved. Wheelchair. No neuropsychological deficit.	Hydrocephalus requiring shunt
Kneyber et al., 2005 [4]												
Patient 1	5	Fall	Acute drop in conscious level, recover and further drop in consciousness at day 7	Basilar	7	Yes	No (first CTA was at day 7 after rebleed)	CTA at day 7	Conservative	Yes	Severe neurological deficits	Stroke
Patient 2	3	Fall	Acute drop in conscious level	Right vertebral	14	Yes	No	MRA at day 14	Surgical occlusion of vertebral	Yes	Severe neurological and neurophysiological deficits	Stroke
Binning et al., 2009 [5]	15	Assault	Acute drop in conscious level	Left PICA	21 months	Yes	Yes, initial CTA negative	Delayed CT and DSA at 21 months	Clipping and bypass	VP shunt	No new deficits	Hydrocephalus requiring shunt
Kaplan et al., 1993 [6]	41	Post partum, presumed traumatic	Head and neck pain left arm and hand paraesthesia	Left vertebral (extracranial)	28	No (LP positive)	No	CTA and then DSA at 4 weeks	Balloon occlusion	No	No new deficits	No
Schuster et al., 1999 [7]												
Patient 1	22	Assault	Acute drop in conscious level	Right PICA	1	Yes	No	DSA at day 1	Clipping	Yes	Living independently with some memory difficulty at 1 year	Hydrocephalus requiring shunt, vasospasm requiring angioplasty
Patient 2	16	Hit with baseball	Acute drop in conscious level	Right PICA	3	Yes	Yes negative initial DSA, DSA at 72 hours positive	DSA at day 3, Day 1 DSA normal	Clipping	Yes	In assisted living placement at 6 months	Hydrocephalus requiring shunt, vasospasm requiring angioplasty
Patient 3	33	Fall	Acute drop in conscious level	Left PICA	1	Yes	No	DSA at day 1	Clipping	Yes	In assisted living placement at 2 years	Hydrocephalus requiring shunt, vasospasm requiring angioplasty, DVT/PE
Sure et al., 1999 [8]	9	Closed head trauma	Hydrocephalus needing EVD after head injury and then further severe headache 3 weeks after initial head injury due to rebleed	Left PICA	21	Yes	No	DSA and MRI at 3 weeks	Clipping (failed coil)	Yes	No deficits	Hydrocephalus requiring shunt
Nishioka et al., 2002 [9]												
Patient 1	20	Direct head trauma during karate	Seizure and reduced consciousness with rebleeds at day 15 and 66	Right PICA	11	Yes	No	DSA at day 11	Too unstable for treatment	Yes	Death	Hydrocephalus and multiple rebleeds
Patient 2	33	Hit head on wall	Headache and nausea and then delayed acute drop in consciousness at day 14 due to rebleed	Left PICA	1	Yes	No	DSA at day 1 and day 14	Clipping	No	No deficits	Rebleed, hydrocephalus requiring shunt
Malek at al, 2000 [10]	42	Direct head trauma during kickboxing	Headache, cranial nerve palsies, meningism with acute drop in consciousness at day 6	Left vertebral	6	Yes	No	DSA at day 6	Coiling	No	No deficits	No
Kim et al., 2014 [1]	51	Fall	Reduced consciousness with further deterioration in consciousness the same day secondary to rebleed	Basilar	1	Yes	No	CTA at day 1	Coiling	No	Death	Rebleed, cerebral swelling
Ong et al., 2010 [11]	3	Fall	Acute drop in consciousness at 2 weeks post injury	Left superior cerebellar artery	14	Yes	No	CTA and DSA at day 14	Coiling	No	Death	_
O;Shaughnessy et al., 2005 [12]	13	Oral gunshot wound	Intact consciousness at presentation, VA injury found on imaging and treated endovascularly. Delayed deterioration in consciousness and headache at day 8 due to bleed from VA TICA	Left vertebral	8	At second presentation after TICA rupture on day 8	No	DSA at day 1 (occlusion) and day 8 (TICA)	Clipping after bleed from new TICA post coiling	Yes	No deficits at 1 year	Hydrocephalus not needing permanent CSF diversion
Cohen et al., 2010 [13]	23	Direct neck trauma during taekwondo	Acute drop in conscious level	Left vertebral	1	Yes	No	DSA at day 1	Endovascular vessel occlusion	Yes	Mild dizziness and ataxia at 1 month	Hydrocephalus not needing permanent CSF diversion
Coulter et al., 2011 [14]	20	Road traffic accident	Acute drop in conscious level	Right vertebral	1	Yes	No	DSA at day 1	Endovascular vessel occlusion	No	No deficits	No
Purgina et al., 2015 [15]	22	Assault	Loss of consciousness with improvement. Acute deterioration in consciousness at day 9 due to rebleed	Left PICA	7	Yes	Yes, day 1 DSA negative, CTA at day 7 positive	DSA at day 1 normal, CTA at day 7 small PICA aneurysm, DSA at day 9 enlarged PICA aneurysm	Too unstable for treatment	No	Death	Rebleed and hydrocephalus
Morard & deTribolet, 1991 [16]	31	Fall	Acute drop in conscious level	Right PICA	10	Yes	No	DSA at day 10	Clipping	Yes	Full recovery	Hydrocephalus requiring shunt
Chiang et al., 2009 [17]	50	Assault	Acute drop in conscious level	Right vertebral	1	Yes	No	DSA at day 1	Clipping	No	Full recovery	No
Meguro et al., 1985 [18]	54	Road traffic accident	Quadriparesis and cranial nerve palsies	Right PICA	6	Yes	No	DSA at day 6 and day 33	Surgical excision	No	Almost independent	No
Quattrocchi et al., 1990 [19]	26	Assault (penetrating)	Acute drop in conscious level and then rebleed after EVD sited	Left superior cerebellar artery	1	Yes	No	DSA at day 1	Too unstable for treatment	Yes	Death	Rebleed
Bell et al., 2010 [20]	24	Penetrating neck injury	Acute drop in conscious level on day 4 post injury	Left PICA	4	Yes	Yes, initial CTA negative, DSA at day 4 and 7 positive	DSA at day 4 and at day 7 which showed enlargement	Coiling	Yes	Almost independent	No
Present case 1	20	Assault	Acute drop in conscious level	Right vertebral	11	Yes	Yes, CTA at presentation negative	Initial CTA negative, died before DSA	Too unstable for treatment	No	Death	Hydrocephalus and rebleed
Present case 2	24	Assault	Acute drop in conscious level	Left PICA	1	Yes	No	CTA and DSA at day 1	Coiling and stenting	Yes	Almost independent	EVD related haematoma
Present case 3	35	Fall	Acute drop in conscious level, recovery and further drop in consciousness at day 7 due to rebleed	Left PICA	7	Yes	Yes negative initial CTA	Initial CTA negative, DSA at day 7	Coiling	Yes	Death	Hydrocephalus, Shunt malfunction

## Discussion

The key message of our case series and review is that SAH secondary to TICA of the posterior circulation is a treatable condition of young patients, which if misdiagnosed or diagnosed late can prove fatal. We highlight the potential for delayed appearance of posterior circulation TICA on imaging and misdiagnosis of these cases as non-traumatic SAH or as “uncomplicated” traumatic SAH with no early and delayed search for an underlying TICA. We also highlight the high incidence of rebleed, hydrocephalus and morbidity associated with this condition.

### Aetiology and pathomechanism

Although trauma remains the most common aetiology of SAH, posterior circulation TICA is rare. It is even more rarely reported as a cause for SAH. TICA is histologically a pseudoaneurysm (TICA is a relatively new terminology and many of the cited reports use the term pseudoaneurysm when discussing this condition). Pathologically, a pseudoaneurysm requires all three layers of the vessel wall to be disrupted and integrity of the vessel is only maintained by associated haematoma or surrounding connective tissue [21]. In true aneurysms the adventitia is preserved. Intracranial vessels, unlike cervical vessels, have a thinner media and no external elastic lamina, making them more vulnerable to injury [22].

The extracranial vertebral arteries are traditionally believed to be more vulnerable to trauma than intracranial vessels owing to their relatively exposed location within the cervical vertebrae. However, our review demonstrates PICA to be the most common vessel involved, in 54 % of cases [23]. Almost one third of cases were in patients aged under 16. The higher proportion of traumatic aneurysms in children may be due to the paediatric cervical spine and craniocervical junction being relatively mobile, exposing vessels to stretching and shearing forces. The overall young age of presentation across this review (mean age 24) and the male preponderance (75 %) [19] also reflects the higher incidence of trauma in young males.

When considering SAH from posterior circulation TICA, the paucity of reported cases may be accounted for by the fact that much of the literature on this condition is in the forensic literature, as it is often rapidly fatal and diagnosed at post mortem. Blunt trauma was the injury mechanism for the majority of cases in this review. In penetrating head injury, primarily seen in the military environment, intracranial vascular injury occurs in over 20 % of patients [20]. What predisposes to TICA formation after traumatic vascular injury in some patients and not others cannot be explained solely by the severity or mechanism of trauma, as there are cases reported after relatively minor injuries. Proposed mechanisms for vascular disruption include rotational head acceleration, hyperextension of the vertebrobasilar vessels and acute severe short-lasting intracranial hypertension from a blow to the carotids [24]. Alterations in local haemodynamics such as flow reversal and transient occlusion were found in an ex vivo study on vertebro-basilar vasculature to be sufficient to induce longitudinal tears in the vessel walls, which may lead to TICA formation. Histological features of the vessel wall, especially the adventitia, have been reported in the forensic literature to distinguish TICA from non-traumatic pathology [25, 26]. In the PICA, the proximal segment and regions close to perforators are reported as vulnerable to trauma, possibly due to the anchoring effect of the perforators and vessel origin leading to shear stress when trauma is inflicted, causing a “tear out”. The posterior cerebral artery (PCA) and superior cerebellar artery (SCA) may be damaged against the rigid tentorial edge [7, 8]. Vessels may be more vulnerable in certain patients due to underlying conditions such as fibromuscular dysplasia and atherosclerosis [7]. Once a TICA has formed, the factors predisposing it to expansion, rupture or regression are not fully understood. There is little evidence available on the proportion of individuals who develop a pseudoaneurysm post trauma that resolves.

### Clinical features

Clinical diagnosis of a traumatic posterior circulation TICA is challenging. If sufficiently large to compress surrounding structures, an unruptured TICA may present with neck pain, lower cranial nerve palsies, cervicomedullary compression syndrome and Horner’s [6]. SAH from a ruptured TICA presents with the same features as SAH from congenital aneurysms, including severe headache, vomiting, seizures, focal deficits, altered consciousness and features related to an intracerebral clot or acute hydrocephalus. In our review, 88 % of patients had an acute drop in conscious level; 46 % had this drop in a delayed manner (hours to 1 week on average), half of these due to rebleed. This suggests three possibilities: first that the TICA had developed in a delayed manner in patients whose first bleed was delayed after the trauma; second, that patients with SAH immediately after trauma had bled from a vascular tear and a delayed rebleed was from a TICA that formed after this (analagous to the well-described iatrogenic complication of pituitary surgery where a traumatic carotid tear leads to a pseudoaneurysm); third, that a TICA was present early after trauma but not detected on vascular imaging and expanded before rebleeding. We found the incidence of rebleed to be almost a quarter of patients (23 %) and hydrocephalus requiring emergency CSF diversion in 61 %. The mortality rate in this review was 27 %. Older series reported the mortality rate for ruptured cranial traumatic TICAs (mainly anterior circulation) to be approximately 30–50 % without treatment and up to 25 % with treatment [27]. In Asari’s 1977 review of 60 cases, the rebleed rate was 45 % and the condition mainly affected young patients (39 % under age 20) [27]. Although the aneurysm location was mainly anterior circulation in Asari et al.’s review, it is evident that the mortality rate has not significantly changed over the last 40 years despite advances in imaging and treatment. This highlights the need to detect the TICA early, reducing the risk of rebleed and allowing treatment whilst it is smaller and technically less complex to treat.

In terms of long-term follow-up, one fifth of patients in this series (19.2 %) could not live independently. Outcome, as with other neurological injuries, is likely to be influenced by both primary and secondary injuries. One method to reduce the risk of primary and secondary injury in these cases is to reduce the rebleed risk. This may be achieved by repeating vascular imaging in patients with cisternal SAH, or a distribution of subarachnoid blood that appears disproportionate to the history, post trauma and no vascular abnormality on admission neurovascular imaging.

### Imaging

In the majority of traumatic SAH, the pattern of blood is focal, cortical and supratentorial, with a clear trauma mechanism. In these circumstances, vascular imaging is seldom performed. In cisternal pattern traumatic SAH and an uncooperative patient, it may be unclear whether the trauma precipitated or preceded an intracranial vascular event. A retrospective series of 130 patients with blunt head trauma and SAH demonstrated that only 8 % of SAH related to head injury resulted from congenital aneurysm rupture [28]. SAH from a traumatic posterior circulation TICA may therefore be initially misdiagnosed as aneurysmal SAH [17]. With a history of trauma and cisternal SAH identified on CT scans, vascular imaging is usually undertaken (CTA and/or DSA) during hospital admission. Owing to the paucity of reported cases, there is no consensus on the optimal imaging modality or timing of imaging. In the absence of a cervical spine fracture, but cisternal SAH associated with trauma, there is variable practice regarding inclusion of the cervical spine in vascular investigations. CTA and magnetic resonance imaging (MRI) of the cervical spine are the most commonly undertaken. Using the knowledge that TICA formation is usually delayed [3, 29] (mean time to diagnosis in our review was 1 week and 85–90 % present in 3 weeks [8]), TICAs exhibit faster growth than berry aneurysms and have a high rebleed risk—5–7 days maybe a reasonable starting point for follow-up imaging. In penetrating head trauma, the mean time to diagnosis of TICA is 10 days, suggesting delayed TICA formation is common to both blunt and penetrating trauma [20]. Angiographic features of posterior circulation TICA include fusiform morphology, not being located at vascular bifurcations, irregular sac morphology, delayed retention of contrast through the late arterial and venous phases, irregular vessel segments proximal or distal to the aneurysm and appearance of a double lumen [3, 30]. It is important to distinguish a TICA from a dissecting aneurysm as the treatment modalities and risks for these differ [9, 31].

### Treatment options

Management of a TICA is challenging [15] because of the fusiform morphology, disrupted and friable vessel walls, and rapid rate of expansion.

Treatment strategies for TICA and dissections include radiological monitoring, antiplatelet/anticoagulation medications, endovascular treatment (coiling, stenting or vessel sacrifice), surgical clipping, trapping and bypass procedures [32]. In the presence of SAH, anticoagulation/antiplatelets are contraindicated, which pushes management towards endovascular and surgical. It may not always be possible to preserve the parent vessel but if there is adequate cross flow, this might be done without neurological deficits [2]. There are no large data sets on outcomes that can be used to identify the optimal treatment modality and therefore management is currently on a case-by-case basis. A recent meta-analysis of 39 studies on endovascular treatment for non-traumatic vertebral dissection demonstrated excellent outcome, but there remains a relatively high risk of treatment-related complications (approximately 10 %) [33]. Although this study is not looking specifically at traumatic dissection or SAH from the dissection, it suggests that treatment-related morbidity is significant. Although vasospasm is well documented to be a complication of traumatic SAH, there is no evidence supporting the use of nimodipine for non-aneurysmal SAH [34, 35].

## Conclusion

In view of the aggressive natural history of posterior circulation TICA, we recommend that CTA of the head and neck vessels be performed for cases presenting with post-traumatic disproportionate cisternal and or third or fourth ventricular SAH. In the event of initial CTA being negative, repeat CTA and if negative DSA should be performed between 5 to 7 days, with a low threshold for further repeat at 10 days if a traumatic dissection is still suspected. Close monitoring for hydrocephalus and vasospasm is required during hospital admission and significant therapy input is likely to be required post discharge from acute care.
